# Implementation of surgical site infection surveillance in 16 health facilities in Sierra Leone

**DOI:** 10.1017/ash.2022.178

**Published:** 2022-05-16

**Authors:** Rugiatu Z. Kamara, Monique Foster, Jamine Weiss, Christiana Conteh

## Abstract

**Background:** Surgical site infections (SSIs) are associated with increased healthcare costs, antibiotic resistance, morbidity, and mortality. In low- and middle-income countries (LMICs), SSIs account for most healthcare-acquired infections (HAIs). In Africa, up to 20% of women who undergo a caesarean section develop a wound infection. Surveillance has been shown to be an essential component in the overall strategy to reduce SSIs. **Methods:** Surgical site infection surveillance is being implemented in 16 health facilities in Sierra Leone, with at least 1 from each of the 5 US Census regions: Eastern, Western, Northern, Northwestern, and Southern. These health facilities were selected based on the availability of a dedicated infection prevention and control (IPC) focal person. Women were observed for 30 days after caesarean section. A standardized surgical safety and surveillance checklist including case definitions and observable criteria (eg, purulent drainage, wound abscess, or intentional reopening) was used. Clinical staff were trained to collect data and to conduct in-person and phone interviews with patients on days 3, 7, and 30 after caesarean section. **Results:** From March 2021 to July 2021, a total of 2,529 women had caesarean sections in 15 health facilities; most occurred in the Northern region (785 of 2,529). Among these 2,529 women, 1,522 (60%) had an SSI surveillance checklist started, and of those 1,522, 632 (42%) had a completed checklist. Health facilities in most of the rural regions, (Eastern, Northwestern, and Southern) had no completed checklists. The overall SSI rate for the 15 health facilities was 3% (70 of 2,529). The Southern region had the highest SSI rate at 50% (35 of 70), but the Western region did not report any SSIs. Of the 70 cases, 49 (70%) were identified through active inpatient surveillance and 21 (30%) were identified through postdischarge surveillance. **Conclusions:** One of the priorities of Sierra Leone’s National IPC Action Plan is to establish HAI surveillance. Surgical site surveillance is an essential component of HAI surveillance and leads to timely identification so infections can be treated quickly. This study was limited by inadequate data collection and patients lost to follow-up after discharge. However, this study illustrates that surveillance leads to the diagnosis of most SSI cases after caesarean section while patients are still hospitalized. Simple yet effective SSI surveillance can be conducted in LMICs to identify and ultimately treat SSI after caesarean section. More support is needed in rural and smaller facilities for better implementation of SSI surveillance in Sierra Leone.

**Funding:** None

**Disclosures:** None

